# Empagliflozin Attenuates Obesity-Related Kidney Dysfunction and NLRP3 Inflammasome Activity Through the HO-1–Adiponectin Axis

**DOI:** 10.3389/fendo.2022.907984

**Published:** 2022-06-17

**Authors:** Tongtong Ye, Jingwen Zhang, Di Wu, Junfeng Shi, Zengguang Kuang, Yuting Ma, Qian Xu, Bing Chen, Chengxia Kan, Xiaodong Sun, Fang Han

**Affiliations:** ^1^ Department of Endocrinology and Metabolism, Affiliated Hospital of Weifang Medical University, Weifang, China; ^2^ Clinical Research Center, Affiliated Hospital of Weifang Medical University, Weifang, China; ^3^ Department of Pathology, Affiliated Hospital of Weifang Medical University, Weifang, China

**Keywords:** Empagliflozin, obesity, kidney disease, NLRP3, HO-1

## Abstract

Empagliflozin (EMPA) is a novel sodium-glucose cotransporter 2 inhibitor (SGLT2i) that produces protective cardiovascular-renal outcomes in patients with diabetes. However, the effects of EMPA on obesity-related kidney disease have not been determined. The heme oxygenase-1 (HO-1)–adiponectin axis is an essential antioxidant system with anti-apoptotic and anti-inflammatory properties. This study explored whether EMPA improves obesity-related kidney disease through regulation of the renal HO-1-mediated adiponectin axis. C57BL/6J mice were assigned to control, high-fat diet (HFD) groups, and EMPA (10 mg/kg) groups. HFD mice showed metabolic abnormality and renal injury, including increased urinary albumin excretion, morphologic changes, and lipid accumulation. EMPA treatment improved metabolic disorders and attenuated lipotoxicity-induced renal injury. Furthermore, EMPA treatment ameliorated renal NLRP3 inflammasome activity and upregulated the HO-1–adiponectin axis. Our findings indicate that EMPA improves obesity-related kidney disease through reduction of NLRP3 inflammasome activity and upregulation of the HO-1–adiponectin axis, suggesting a novel mechanism for SGLT2i-mediated renal protection in obesity.

## Introduction

The prevalence of obesity, an important public health problem, has substantially increased over the past 30 years (1). This increased prevalence has implications for various complications, including renal damage known as obesity-related kidney disease (OKD) (2). Generally, the onset of OKD is unnoticed; most patients initially have no obvious clinical symptoms, with the exception of microalbuminuria identified during physical examination. In patients with obesity, hyperfiltration often occurs as a compensatory mechanism for the increased metabolic demands. This causes damage to renal structure and function, leading to OKD and the potential for end-stage kidney disease (3, 4). The pathogenesis of OKD usually involves high metabolic demand, insulin resistance, chronic inflammation, and disordered lipid metabolism (5). However, the mechanisms by which obesity contributes to the induction or progression of OKD have remained unclear.

Heme oxygenase-1 (HO-1) is an inducible enzyme/protein that catalyzes the oxidative degradation of heme to bilirubin (6). HO-1 can sense and respond to various metabolic alterations, including oxidative and inflammatory stress. Increased HO-1 expression is considered a promising therapeutic method for metabolic disease alleviation through the regulation of cellular function and pathophysiology (7). Notably, HO-1 may mediate beneficial effects by enhancing adiponectin secretion; this pathway is known as the HO-1–adiponectin axis (8–10). The activation of this axis in obese animal models may suppress inflammatory cytokine activity and protect against OKD (8, 11). Adiponectin is mainly secreted from adipose tissue that has potent anti-inflammatory, antiatherogenic, and vasoprotective properties (12). Circulating adiponectin levels are usually decreased in obesity and metabolic disease. Adiponectin therapy has glucose-lowering effects and can ameliorate insulin resistance (13). Several studies have reported favorable results of adiponectin treatment in metabolic disease (14, 15). Notably, adiponectin is also produced in non-adipose tissue, particularly in the kidney (e.g., in glomerular endothelial cells and tubular cells) (16). However, there is a need to identify the mechanism by which the renal HO-1–adiponectin axis affects OKD.

Empagliflozin (EMPA), a new oral glucose-lowering drug, selectively acts on sodium-glucose cotransporter-2 inhibitor (SGLT2i) receptors in proximal kidney tubule epithelial cells; it inhibits sodium-glucose cotransporters to reduce blood glucose (17). The most direct effects of SGLT2i therapy include the restoration of tubule feedback and reduction of both oxidative stress and inflammation; these effects have renoprotective and cardioprotective outcomes (18). Although SGLT2i therapy improves diabetic nephropathy outcomes, no study has investigated whether OKD can be alleviated by SGLT2i therapy in patients or animals with obesity. This study examined whether EMPA could improve OKD through the HO-1–adiponectin axis in high-fat diet (HFD)-induced obese mice.

## Materials and Methods

### Experimental Animals

Four-week-old male C57BL/6J mice were purchased from Jinan Pengyue Laboratory (China). All mice were randomly assigned to normal control (NC), HFD, and HFD-EMPA (HFD-E) groups. Mice in the NC group were fed a regular diet, while mice in the other groups were fed an HFD (45% fat, 530 kcal/100 g; Fanbo Biotechnology, Wuxi, China). After they had received an HFD for 24 weeks, mice in the HFD-E group were administered EMPA (10 mg/kg/day) (Boehringer Ingelheim) by oral gavage for another 8 weeks. Body weight and body composition analysis were measured weekly (Bruker Minispec LF50, Germany). After 32 weeks of feeding, glucose tolerance assessment, insulin resistance assessment, and 12-h urine collection were performed; mice were sacrificed 1 week later. The study protocol was approved by the Animal Ethics Committee of Weifang Medical University.

### Oral Glucose Tolerance Test and Insulin Tolerance Test

After they had been fasted for 6 h, the mice were administered 2 mg/g glucose by oral gavage (oral glucose tolerance test) or 0.75 U/kg regular insulin (diluted in saline) by intraperitoneal injection (insulin tolerance test). Tail venous blood were collected for assessment with a blood glucometer (On Call EzIII, China) at various time points.

### Biochemical Assays

Plasma triglycerides (TG) and free fatty acid (FFA) concentrations were measured using commercial test kits (BC0625 and BC0596, Solarbio, China). Urinary albumin was measured using an enzyme-linked immunosorbent assay (CEB028Mu, Cloud-Clone Corp, China). Urinary creatinine was measured using a test kit from Jiancheng (Nanjing, China).

### Immunofluorescence

For the detection of NLR family, pyrin domain containing 3 (NLRP3) expression patterns, frozen tissue blocks were cut into 5-μm sections. After they had been washed with phosphate-buffered saline, tissues were fixed with 4% paraformaldehyde solution for 10 min, permeabilized with 0.3% Triton-X 100 (TB8200, Solarbio, China) for 20 min, blocked with 1% bovine serum albumin, then followed by incubated with NLRP3 antibody (#15101, Cell Signaling Technology) (1:200) overnight. Subsequently, the tissues were incubated with goat secondary antibody (1:600; ab150077, Abcam) at room temperature, then incubated with 4′,6-diamidino-2-phenylindole (DAPI, 1:200) for 10 min. Finally, NLRP3 expression patterns were photographed using a microscope (Zeiss, Germany).

### Histopathological Analysis

Kidney tissues were separated in cooled saline, then immediately fixed in 4% paraformaldehyde and embedded in paraffin. Five-millimeter sections were cut from paraffin blocks, then stained with hematoxylin and eosin for histopathology analysis. Kidney tubular interstitial fibrosis was evaluated by Masson’s trichrome and Picrosirius Red staining, while lipid accumulation was assessed by Oil Red O staining. Photographs were acquired using Motic Digital Pathology Solution (Easyscan, Motic, China).

### Transmission Electron Microscopy

Mouse kidney tissues were fixed in sodium cacodylate buffer. Fixed tissues were trimmed to 1-mm^3^ cubes for embedding. Sixty-nanometer-thick sections were cut using an ultramicrotome (Leica UC7, Leica, Germany); the sections were placed in cuprum grids. The cuprum grids were then imaged *via* transmission electron microscopy (HT7800/HT7700, Hitachi, Japan).

### Western Blotting

Kidney tissue was ground with a manual homogenizer and homogenized in cold protease inhibitor lysis buffer for 30 min. It was then centrifuged at 12000 rpm for 10 min at 4°C; the supernatant was subjected to protein quantification *via* bicinchoninic acid assay. The samples were separated *via* 12% sodium dodecyl sulfate–polyacrylamide gel electrophoresis, transferred to polyvinylidene fluoride membranes, and incubated with antibodies to the following proteins: HO-1 (#43966, Cell Signaling Technology); Adiponectin (ab181281, Abcam) and β-actin (66009-1-Ig, Proteintech).

### Reverse Transcription Polymerase Chain Reaction Analysis

Total RNA from the left kidney was isolated and extracted using TRIzol Reagent (Invitrogen). Then, the extracted RNA was reverse-transcribed using a PrimeScript™ RT Reagent Kit with gDNA Eraser (#RR047A, TaKaRa). TB Green^®^ Premix Ex Taq™ II (#RR820A, TaKaRa) was used for quantitative polymerase chain reaction analysis. Relative changes in expression levels of amplified genes were determined using the comparative cycle threshold (Ct) method (i.e., 2^-ΔΔCt^). Relative expression levels of the interleukin (IL)-1β, IL-6, IL-18, and NLRP3 genes were normalized to the expression of β-actin. The primers used in this study are shown in **Table 1**.

**Table 1:** The primers used in the study.

Gene	Primer sequence (5’→3’)	
**β-actin**	F: GGCTGTATTCCCCTCCATCG	R: CCAGTTGGTAACAATGCCATGT
**IL-1β**	F: GCAACTGTTCCTGAACTCAACT	R: ATCTTTTGGGGTCCGTCAACT
**IL-18**	F: GACTCTTGCGTCAACTTCAAGG	R: CAGGCTGTCTTTTGTCAACGA
**IL-6**	F: CTGCAAGAGACTTCCATCCAG	R: AGTGGTATAGACAGGTCTGTTGG
**NLRP3**	F: ATTACCCGCCCGAGAAAGG	R: TCGCAGCAAAGATCCACACAG

### RNA-Seq and Data Analysis

The RNA sequence and data analysis were prepared as previously described by our group (19). Differentially expressed genes (DEGs) were identified using limma packages in R 4.0.3 with the default parameters at (logFC) > 1, P.Value < 0.05 for the groups. And Gene Ontology (GO) and Kyoto Encyclopedia of Genes and Genomes (KEGG) analyses of DEGs were conducted using the clusterProfiler package in R based on the criteria: Adjusted.P.Value<0.01.

### Statistical Analysis

All statistical analyses were carried out using GraphPad Prism 8. The results are shown as means ± standard errors of the mean. One-way or two-way ANOVA and Turkey’s test were utilized as appropriate. Differences were considered statistically significant based on the following setting: P<0.05.

## Results

### EMPA Treatment Decreased Weight Gain, Fat Mass, and Fat%, While Normalizing Glucose Intolerance, in HFD Mice

**Figure 1:**
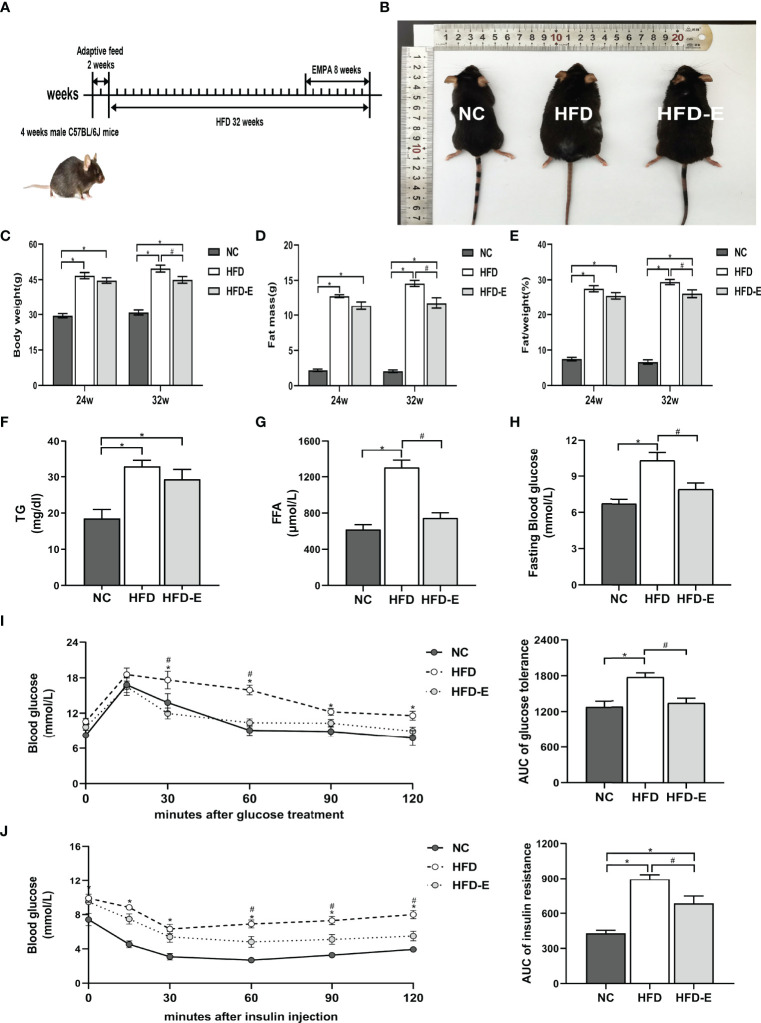
EMPA reduced body weight and glycolipid metabolism. **(A)** 4-week-old male mice were fed HFD for 24 weeks and then treated with EMPA for another 8 weeks. **(B)** Morphology of mice. **(C)** Body weight. **(D)** Body fat mass. **(E)** Body fat mass%. **(F)** TG levels. **(G)** FFA levels. **(H)** Fasting blood glucose levels. **(I)** Oral glucose tolerance test and area under curve (AUC) of glucose tolerance. **(J)** Insulin tolerance test and AUC of insulin tolerance. Data are means ± SEM, n = 6/group, *P < 0.05 vs. NC; ^#^P < 0.05 vs. HFD.

After 32 weeks of HFD administration, HFD mice exhibited significant morphological changes compared with NC mice (**Figures 1A, B**). Specifically, HFD mice had significantly increased body weight (49.67 ± 1.48 g vs. 30.82 ± 1.08 g, P<0.05), fat mass (14.56 ± 0.43 g vs. 2.05 ± 0.18 g, P<0.05), and fat/weight% (29.37 ± 0.75% vs. 7.74 ± 0.27%, P<0.05), compared with NC mice. However, EMPA treatment significantly decreased the final body weight (44.87 ± 1.42 g vs. 49.67 ± 1.48 g, P<0.05), fat mass (11.75 ± 0.78 g vs. 14.56 ± 0.43 g, P<0.05), and fat/weight% (26.08 ± 1.09% vs. 29.37 ± 0.75%, P<0.05), compared with HFD alone (**Figures 1C–E**). To further explore the lipid metabolism profiles, we measured levels of serum TG and FFA. As expected, these indicators were remarkedly increased in the HFD mice, compared with NC mice (TG: 32.87 ± 1.69 mg/dL vs. 18.51 ± 2.49 mg/dL, P<0.05; FFA: 1303.00 ± 81.14 μmol/L vs. 618.60 ± 52.12 μmol/L, P<0.05); EMPA significantly decreased FFA (746.30 ± 56.59 μmol/L vs. 1303.00 ± 81.14 μmol/L, P<0.05) but had no beneficial effects on TG (29.31 ± 2.71 mg/dL vs. 32.87± 1.69 mg/dL, P>0.05), compared with HFD alone (**Figures 1F, G**). Furthermore, compared with NC mice, HFD mice showed higher fasting glucose levels (10.32 ± 0.66 mmol/L vs. 6.73 ± 0.37 mmol/L, P<0.05) and impairments of both glucose tolerance and insulin tolerance; these alterations were mitigated by EMPA treatment (P<0.05) (**Figures 1H–J**), suggesting that EMPA could alleviate HFD-induced metabolic disorders.

### EMPA Treatment Decreased Kidney Injury in HFD Mice

**Figure 2:**
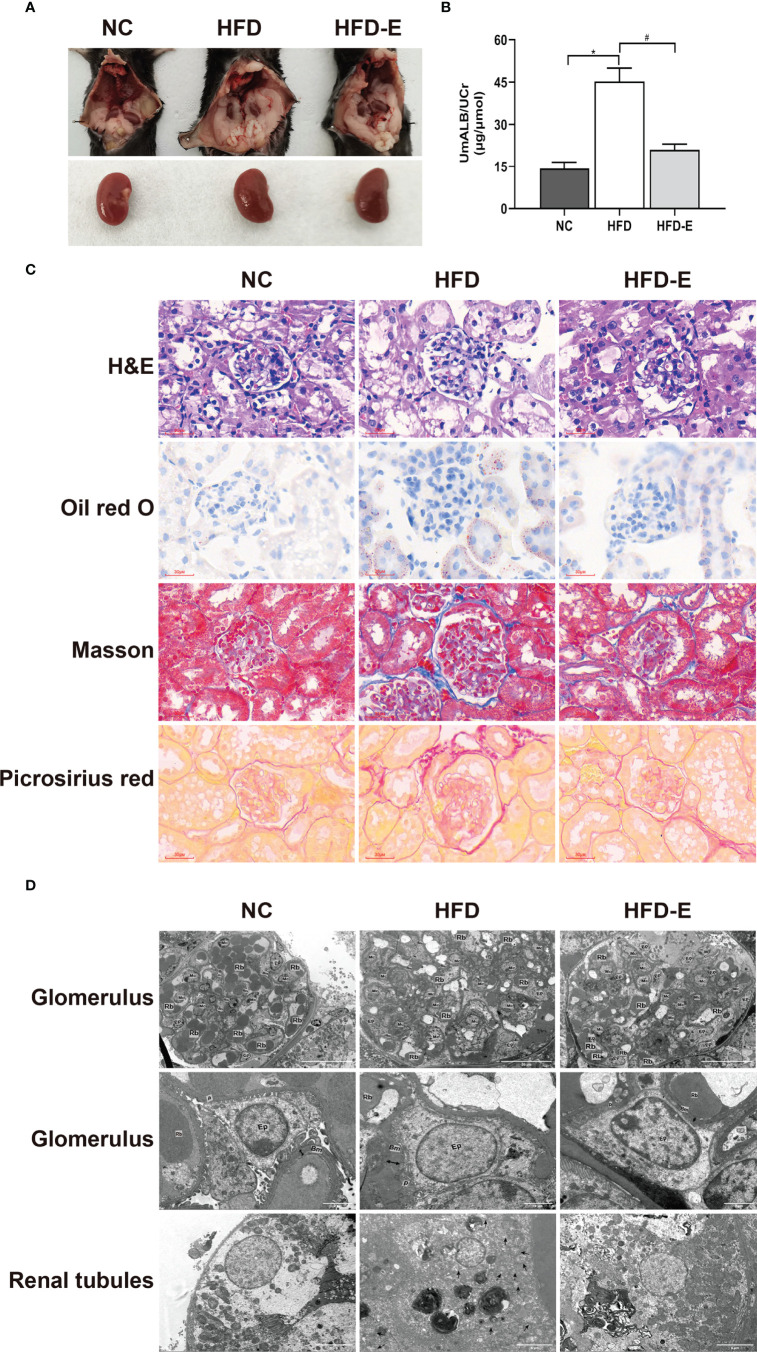
EMPA improved kidney dysfunction and morphologic change. **(A)** Morphology of mice kidney. **(B)** The ratio of urinary albumin to creatinine (n= 6/group). Data are means ± SEM. *P < 0.05 vs. NC; ^#^P < 0.05 vs. HFD. **(C)** H&E, Oil red O, Masson trichrome and picrosirius red staining. Scale bar = 30 µm. **(D)** TEM images of glomerular and tubular structures. Scale bar = 20 µm, 2 µm and 5 µm. BM, basement membrane; Ep, epithelial cells; Rb, red blood cell; Mc, mesangial cells; P, podocyte; Double arrow, basement membrane thickness; * lipid drops; ↑ Damaged mitochondria.

Urinary albumin assessment and histopathology techniques were used to observe renal function. The ratio of urinary albumin to creatinine was higher in HFD mice than in NC mice (45.24 ± 4.71 μg/μmol vs. 14.26 ± 2.28 μg/μmol, P<0.05); EMPA treatment decreased this ratio compared with HFD alone (21.01 ± 1.99 μg/μmol vs. 45.24 ± 4.71 μg/μmol, P<0.05) (**Figure 2B**). HFD mice showed substantial glomerular hypertrophy and renal tubular lumen enlargement in hematoxylin and eosin staining analyses; they also exhibited considerable lipid deposition in Oil Red O staining analyses of renal tubules. Furthermore, HFD treatment induced significant renal fibrosis, compared with NC treatment, in Masson’s trichrome and Picrosirius Red staining. Contemporaneously with these changes, the mitochondria swell and rupture, crest disorder, and increased lipid droplets accumulation in the transmission electron microscope in HFD mice. These morphological alterations were partially reversed by EMPA treatment, indicating that EMPA exerts renoprotective effects in HFD mice (**Figure 2**).

### Kidney Transcriptomic Analyses Revealed Novel EMPA-Induced Pathways in HFD Mice

**Figure 3:**
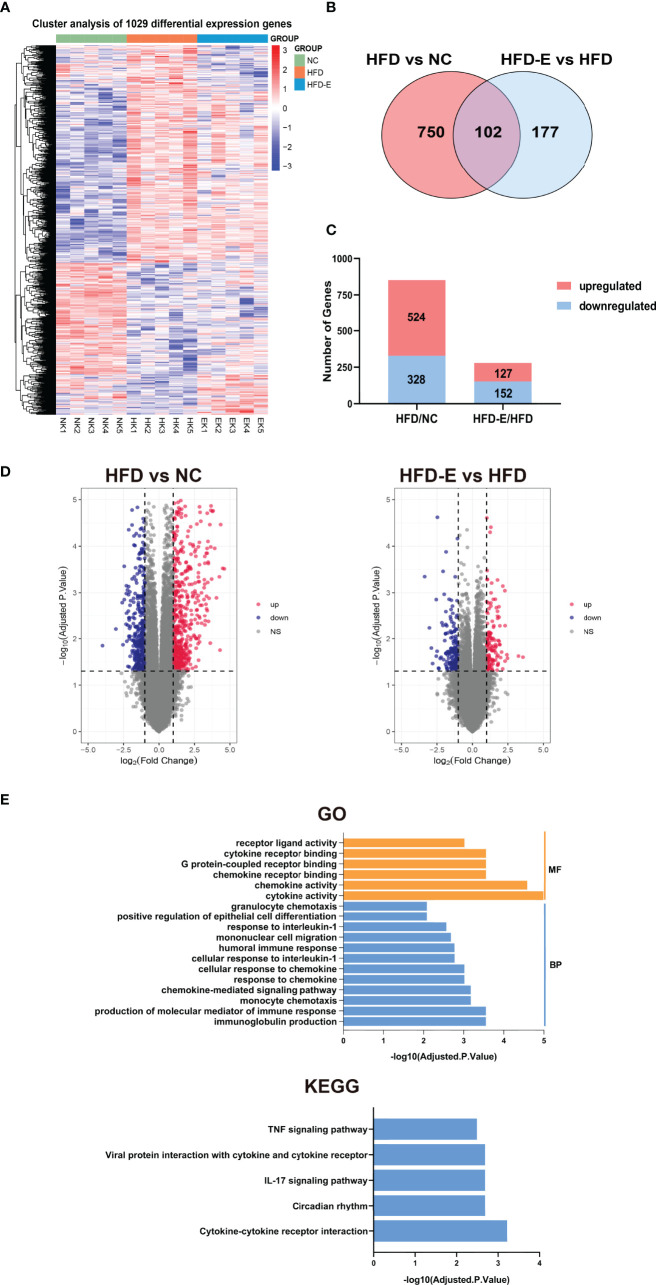
Kidney tissue were collected and then subjected to RNA-seq analysis. **(A)**The DEGs of HFD vs. NC and HFD-E vs. HFD. **(B)**. DEGs associated with HFD vs. NC and HFD-E vs. HFD (light red and blue). **(C)** The number of upregulated and downregulated genes from the HFD vs. NC, and HFD-E vs. HFD in the mice renal genome. **(D)** Volcano plot for the distribution of DEGs between the HFD vs. NC and HFD-E vs. HFD. Blue represents a down-regulation in expression, red represents upregulation and gray represents no significance compared to control. **(E)** Main GO terms and KEGG pathways based on shared DEGs between HFD vs. NC and HFD-E vs. HFD.

To identify potential mechanisms by which EMPA alleviates OKD, three groups of kidneys were subjected to transcriptome profiling. These samples were divided into three groups (NC, HFD, HFD-E), and limma package in R 4.0.3 were used to screening DEGs. DEGs were identified using two comparisons: HFD/NC, and HFD-E/HFD based on (logFC) > 1, P.Value < 0.05. We identified 1029 DEGs union in three groups (**Figure 3A**), 852 DEGs in comparing HFD vs. NC, 279 DEGs in the HFD-E vs. HFD groups, both of which 102 DEGs shared in HFD vs. NC and HFD-E vs. HFD (**Figure 3B**). Additionally, we identified DEGs for which expression levels were altered or reversed by EMPA. In total, 852 DEGs were detected in the HFD vs. NC comparison, of which 524 were upregulated and 328 were downregulated; 279 DEGs were detected in the HFD-E vs. HFD comparison, of which 127 were upregulated and 152 were downregulated. All DEGs were depicted using bar plots and volcano diagrams (**Figures 3C, D**). These DEGs shared in HFD-E vs. HFD and HFD vs. NC were enriched in GO and KEGG categories associated with cytokines and chemokines based on the criteria: Adjusted.P.Value<0.01 (**Figure 3E**).

### EMPA Treatment Decreased NLRP3 Inflammasome Activity in HFD Mice

**Figure 4:**
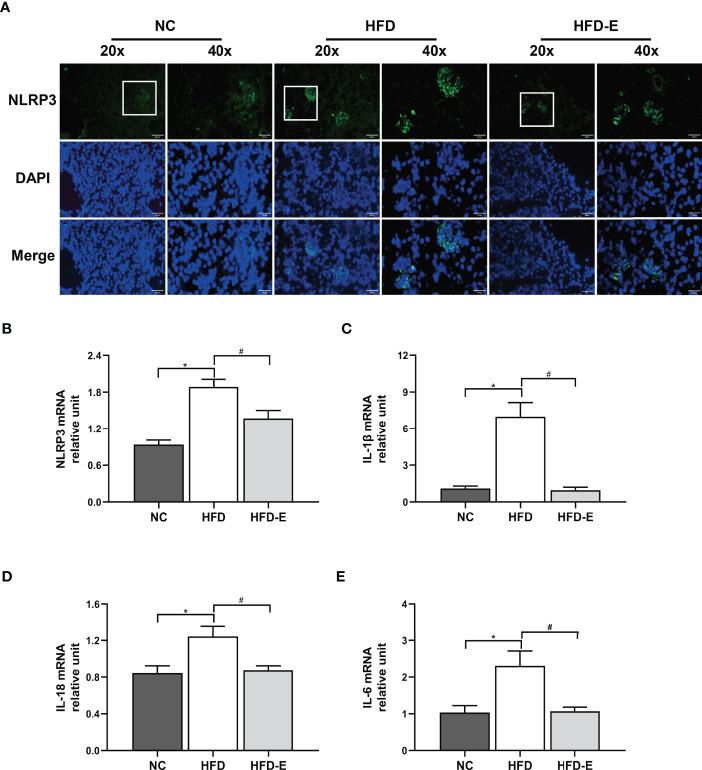
EMPA attenuated the NLRP3 inflammasome. **(A)** Immunofluorescence of NLRP3 inflammasome. Scale bar = 20 µm and 10 µm. **(B–E)** mRNA levels of NLRP3, IL-1β, IL-6, IL-18 (n = 6/group). Data are means ± SEM. *P < 0.05 NC; ^#^P < 0.05 vs. HFD.

Immunofluorescence staining to quantify the protein expression of NLRP3 in mouse kidney tissue revealed significantly elevated expression in HFD mice, compared with NC mice; EMPA treatment reversed this expression pattern (**Figure 4A**). Additionally, we analyzed the transcription levels of NLRP3 and its related genes. Consistent with the immunofluorescence staining results, HFD induced increased transcription of NLRP3, IL-6, IL-1β, and IL-18; these alterations were reversed by EMPA treatment **(**P<0.05; **Figures 4B–E**).

### EMPA Treatment Induced HO-1–Adiponectin Axis Activity in HFD Mice

**Figure 5:**
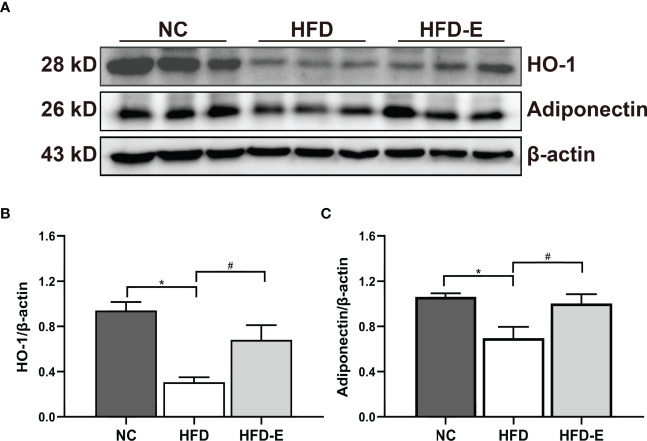
EMPA induced HO-1-adiponectin protein expressions. **(A–C)** Immunoblotting analysis and quantification of HO-1 and adiponectin. n = 6/group; Data are means ± SEM. *P< 0.05 vs. NC; ^#^P < 0.05 vs. HFD.

To verify whether the renal HO-1–adiponectin axis is involved in OKD, we detected the levels of these proteins in kidney tissue; both were significantly decreased in HFD mice. Importantly, EMPA reversed this expression pattern through significant upregulation of HO-1–adiponectin levels (**Figure 5**).

## Discussion

This study investigated the effect of EMPA treatment on OKD in obese mice. We found that HFD induced clear metabolic abnormality and renal injury, accompanied by downregulation of the HO-1–adiponectin axis and enhancement of NLRP3 inflammasome activity. However, EMPA treatment reduced renal injury and NLRP3 inflammasome through activation of the HO-1–adiponectin axis. This study reveals a novel protective role for EMPA in OKD, with a potential underlying mechanism.

Obesity is an increasing public health problem that leads to metabolic syndrome and increased vascular complications, including OKD. Our HFD treatment induced a metabolic syndrome-like phenotype in mice, which included increased body weight and fat. We also observed hyperglycemia, hyperlipidemia, and impaired glucose tolerance in HFD mice. These pathological abnormalities alter metabolic homeostasis and exacerbate kidney damage, as indicated by increased levels of urinary albumin. Furthermore, HFD induced substantial pathological changes, including glomerular hypertrophy, tubule lumen enlargement, renal fibrosis, and mitochondrial injury. Oil Red O staining and transmission electron microscopy demonstrated that lipid droplet accumulation increased in HFD mice. These results indicated that HFD-induced lipotoxicity and associated metabolic abnormalities lead to renal injury, consistent with our previous findings (2, 20).

EMPA is an SGLT2i with cardioprotective, renoprotective, and glucose-lowering effects in diabetic patients. Lu et al. (21) found that EMPA can modulate myocardial contractility; it can also attenuate ischemia and reperfusion injury. Furthermore, EMPA alleviated cardiac inflammation and energy depletion *via* AMPK activation; it exhibited a renoprotective effect by enhancing endogenous ketone body-induced inhibition of mTORC1 (22, 23). Notably, Li et al. reported that EMPA could inhibit epithelial-mesenchymal transition and aberrant glycolysis in proximal tubules, thus protecting renal function (24). Furthermore, EMPA reduces metabolic derangements and restores altered tubule-glomerular feedback, protecting against diabetes-induced cardiorenal injury (25). Our findings demonstrated that EMPA had robust mitigating effects on metabolic and pathophysiological abnormalities in HFD-induced renal injury.

To further elucidate the mechanism by which EMPA protects OKD, we analyzed the mouse kidney transcriptome by RNA-Seq and found 102 DEGs shared in three groups. GO and KEGG enrichment showed that these DEGs were mainly enriched in cytokines, chemokines, and tumor necrosis factor signaling pathways, all of which were association with inflammation process. We discovered that HFD affects inflammatory processes; EMPA can attenuate these processes.

The NLRP3 inflammasome is an important component of pathological inflammation that can trigger local and systemic inflammation (26); it has crucial roles in various diseases (e.g., autoimmunity, diabetes, and cardiovascular disease). The NLRP3 inflammasome is also activated in both acute and chronic kidney disease in mice and humans (27–29). Activation of the NLRP3 inflammasome and subsequent excess production of IL1β, IL-6, and IL18 lead to exacerbation of kidney injury (30). The NLRP3 inflammasome participates in host–pathogen interactions; it recruits and activates pro-inflammatory proteases. Therefore, treatments targeting the NLRP3 inflammasome, the center of inflammatory response, may be useful for the management of various inflammation-related diseases. In the present study, immunofluorescence and RNA-Seq analyses indicated that EMPA treatment attenuated NLRP3 inflammasome activity and inflammation-related biological processes, but the detailed mechanism requires further exploration.

HO-1 is a rate-limiting enzyme that catalyzes heme degradation, with important anti-inflammatory and anti-oxidative properties; it is mainly synthesized by the spleen and “visceral adipose tissue macrophages (31). HO-1 has been shown to reduce NLRP3 inflammasome activity in mice (32); it also reduces visceral fat accumulation, normalizes metabolic profiles, and prevents obesity, thereby reducing cardiovascular and renal complications (7, 33–35). Notably, these beneficial effects were partly mediated through impacts on the adiponectin-dependent pathway (36, 37). Adiponectin is mainly secreted by white adipose tissue; however, its levels are usually lower in the context of obesity and metabolic syndrome, despite adipose accumulation (38). A lower adiponectin level is inversely associated with insulin resistance. Adiponectin has various beneficial effects and modulates many metabolic processes, including anti-atherosclerotic and anti-inflammatory effects (39). We previously showed that HO-1 induction could increase serum adiponectin, thus reducing urinary albumin levels and protecting against OKD by improving endothelial dysfunction (8). These results indicate that HO-1 activation may be a useful treatment for obesity-related renal damage. However, no studies have reported whether renal HO-1 and adiponectin participate in OKD. Here, we found that both of them in kidney tissue were decreased after HFD induction. However, EMPA treatment could increase these levels, indicating the HO-1–adiponectin axis was activated by EMPA. In addition, HO-1 overexpression may protect the D-Galactosamine and lipopolysaccharide-induced hepatic malfunction through suppression of the NLRP3 (40). Adiponectin could inhibit NLRP3 inflammasome activation in nonalcoholic steatohepatitis or cerebral ischemia-reperfusion injury (41, 42). Thus, HO-1 and adiponectin are implicated in NLRP3 inflammasome activation. These findings support our hypothesis that EMPA treatment increased HO-1–adiponectin axis activity and decreased NLRP3 inflammasome activity.

## Conclusion

In conclusion, our study demonstrated that EMPA can protect against OKD by activating the HO-1–adiponectin axis and reducing NLRP3 inflammasome activity in HFD mice. Kidney transcriptome analysis revealed that EMPA affects essential genes closely associated with inflammation. Our findings provide new knowledge concerning the mechanism by which EMPA exhibits protective effects in OKD.

## Data Availability Statement

The data presented in the study are deposited in the NCBI repository, accession number BioProject ID: PRJNA835250.

## Ethics Statement

The animal study was reviewed and approved by Animal Ethics Committee of Weifang Medical University.

## Author Contributions

TY and JZ collected the data, conducted the analysis, and drafted the manuscript. XS and FH designed the entire study and revised the manuscript. Other authors participated in the data collection and analysis.

## Funding

This work was supported by the National Natural Science Foundation of China (81870593, 82170865), Natural Science Foundation of Shandong Province of China (ZR2020MH106), Shandong Province Higher Educational Science and Technology Program for Youth Innovation (2020KJL004), Shandong Province Medical and Health Science and Technology Development Project (202003060396), and Quality Improvement of Postgraduate Education in Shandong Province (SDYAL19156).

## Conflict of Interest

The authors declare that the research was conducted in the absence of any commercial or financial relationships that could be construed as a potential conflict of interest.

## Publisher’s Note

All claims expressed in this article are solely those of the authors and do not necessarily represent those of their affiliated organizations, or those of the publisher, the editors and the reviewers. Any product that may be evaluated in this article, or claim that may be made by its manufacturer, is not guaranteed or endorsed by the publisher.
