# Supplementary material for: Empagliflozin Attenuates Obesity-Related Kidney Dysfunction and NLRP3 Inflammasome Activity Through the HO-1–Adiponectin Axis
Source: Front Endocrinol (Lausanne). 2022 Jun 17;13:907984. doi: 10.3389/fendo.2022.907984 (PMC9248377; doi:10.3389/fendo.2022.907984)
Supplement: Supplementary file 3 [file Table_3.docx]

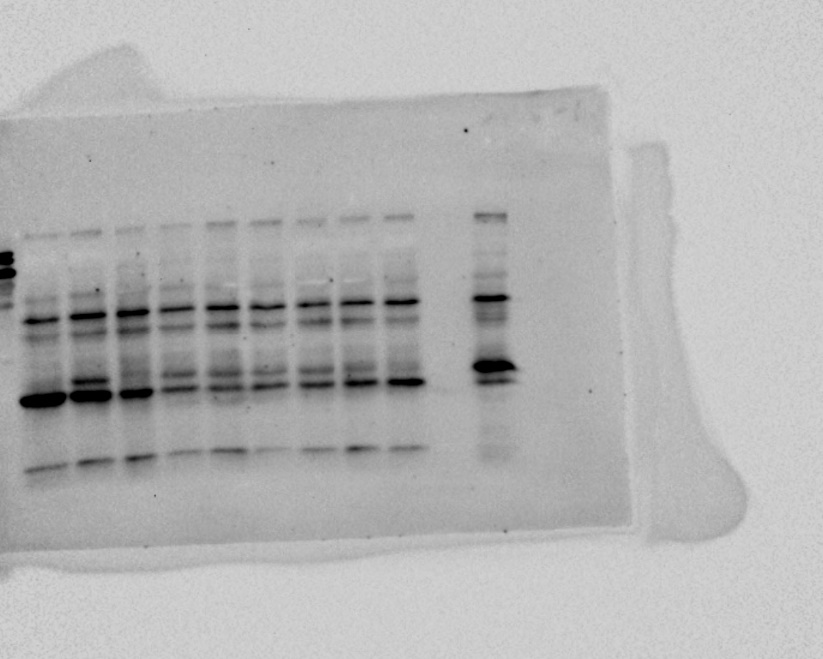


Full unedited gel/blot for Figure5A, HO-1


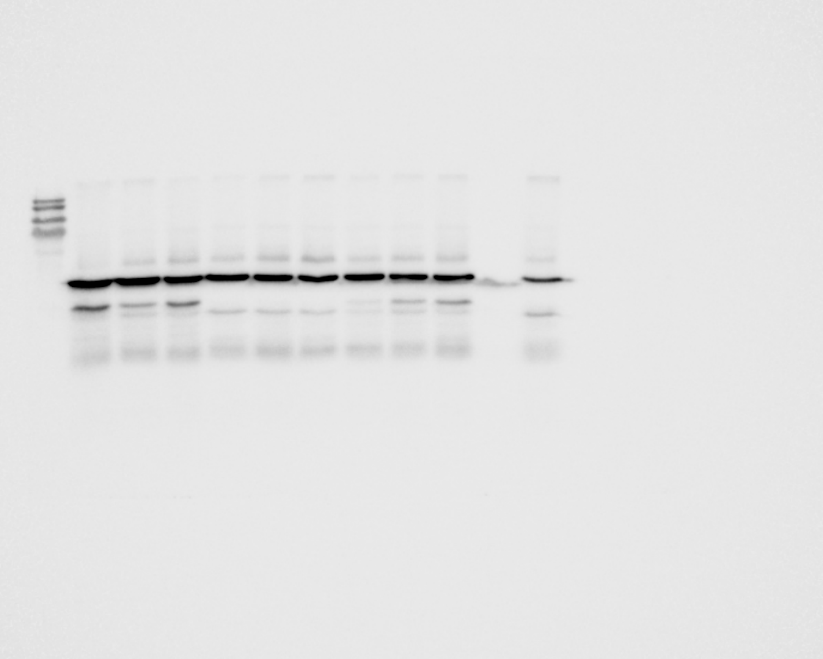


Full unedited gel/blot for Figure5A, β-actin


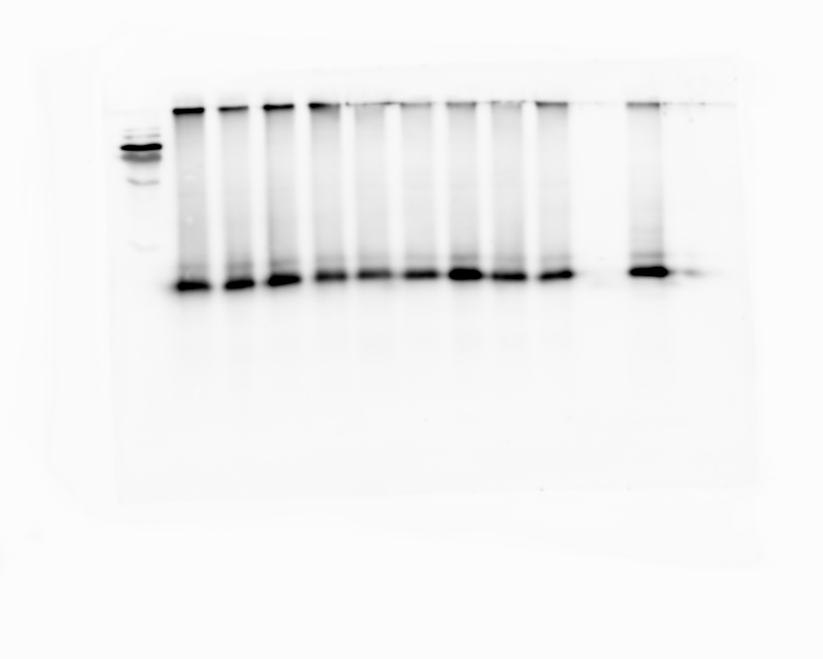


Full unedited gel/blot for Figure5A, Adiponectin
